# Mitigating risk in psychiatric hospital care for older adults by identifying adverse events with Global Trigger Tool for psychiatric patients

**DOI:** 10.1177/09246479251409028

**Published:** 2026-01-11

**Authors:** Arne Okkenhaug, Eivind Aakhus, Guro F. Giskeødegård, Bodil J. Landstad, Ellen T. Deilkås

**Affiliations:** 1 Department of the Director`s Staff, Levanger Hospital, Nord-Trøndelag Hospital Trust, Levanger, Norway; 2 87460 Vestfold Hospital Trust, the Norwegian National Centre for Ageing and Health, Tønsberg, Norway; 3 Department of Public Health and Nursing, Faculty of Medicine and Health Sciences, NTNU, Trondheim, Norway; 4 Department of Human Sciences, Division of Public Health Sciences, Mid Sweden University, Östersund, Sweden; 5 Unit of Research, 59598 Östersund Hospital, Östersund, Sweden; 6 Health Services Research Department, 60483 Akershus University Hospital, Lørenskog, Norway

**Keywords:** adverse event, mental health, patient safety, older adult, psychiatry, health care quality, aged, inpatient, Global Trigger Tool

## Abstract

**Background:**

Older adults receiving psychiatric care are at greater risk of adverse events (AEs) than younger patients. This reflects broader vulnerabilities, including marginalization, complex health needs, and frequent transitions between care settings. It is therefore necessary to investigate AE risk in this population and to validate a previous version of the Global Trigger Tool – Psychiatry (GTT-P), originally developed for the general psychiatric population, for use with older psychiatric patients.

**Objective:**

To apply the Norwegian version of GTT-P in psychiatric care for older adults, to identify the prevalence of AEs in this subpopulation.

**Methods:**

A retrospective cohort study was conducted by reviewing medical records of 184 patients aged 65+ admitted to a psychiatric hospital between 2022 and 2023. All patients who did not opt out were included.

**Results:**

AEs were identified in 10.9% of patients. Triggers related to compulsory treatment and medication significantly increased AE risk. No AEs occurred without associated triggers. Of the AEs identified, 63% were considered avoidable.

**Conclusions:**

This study demonstrates the utility of GTT-P in detecting AEs in older psychiatric patients. Specific clinical triggers were significantly associated with AEs. Preventive strategies and improved care coordination are essential to reduce avoidable harm and enhance patient safety in psychiatric care.

## Introduction

Older adults receiving psychiatric care are at greater risk of adverse events (AEs) than younger patients. This reflects broader vulnerabilities, including marginalization, complex health needs, and frequent transitions between care settings.^1–6^ Suicide rates in the elderly population are high, and patient safety incidents among frail psychiatric patients often go underreported or insufficiently studied.^2,7,8^

Despite increased attention to patient safety in somatic health care, systematic approaches to AE detection in psychiatry—especially among older adults—remain limited.^9–12^ Previous research has mostly emphasized single incident types such as medication errors or falls, leaving gaps in understanding the full scope of safety challenges in this demographic.^9,13–15^

The Global Trigger Tool (GTT) was designed to review random samples of medical records and generate data on the frequency and types of AEs in somatic care.^10^ The GTT has become an internationally recognized and standardized procedure for measuring AEs among adult patients in non-psychiatric hospital admissions,^12,16^ also in Norway.^17^ It is based on a list of triggers with potential to identify specific issues in patient records that suggest a higher probability of an AE.

Internationally, adaptations of the GTT for psychiatric care have emerged in countries such as Sweden and Singapore.^18,19^ Building on these developments, Norwegian psychiatric hospitals have begun exploring the use of GTT-P (Global Trigger Tool – Psychiatry), though empirical data remains scarce—particularly for the older patient population.^20^ In a previous study based on a small general psychiatric population, only 15% of participants were over the age of 60.^21^ It is therefore necessary to investigate AE risk in this population and to validate a previous version of the GTT-P, originally developed for the general psychiatric population, for use with older psychiatric patients.

The aim of this study was to apply the Norwegian version of GTT-P in psychiatric care for older adults, to identify the prevalence of AEs in this subpopulation.

## Methods

### Starting point for the study

The adaptation and validation of the GTT for psychiatric care in a Norwegian context, based on the Swedish version, is previously described by Okkenhaug et al.^21,22^ In this framework, an AE is defined as an accidental or unintended incident occurring in healthcare or services that necessitates further monitoring, treatment or hospitalization, or results in a fatal outcome not attributable to the patient’s illness.^20^ In psychiatric settings, AEs may involve both physical and psychological harm, with mental harm potentially occurring independently of physical injury. Examples include neuroleptic malignant syndrome, suicide, and incidents such as unnecessary deprivation of liberty or exposure to violence. A distinction is made between avoidable and unavoidable AEs,^18^ where avoidable AEs are those that could have been prevented through appropriate care, such as timely diagnosis or the presence of a treatment plan. AEs that can retrospectively be clearly linked to a failure, meaning that assessment, intervention, or treatment was delayed or entirely absent, should be considered avoidable.

The severity of the AEs was rated according the seven-point scale (C-I) based on the National Coordinating Council for Medication Error Reporting and Prevention (NCC-MERP) Index.^23^ We included categories for AEs that could lead to harm.

The GTT-P offers a comprehensive list of triggers that may be evident in patient records and could suggest a higher probability of an AE.^20^ In the GTT-P, a “trigger” refers to an indicator identified during the review of a patient record that serves as a basis for detecting potential AEs, although it is not considered an AE itself. Examples of triggers for potential AEs include readmission within 30 days and insufficient clinical documentation, such as the absence of a treatment plan.

In the Norwegian version of the GTT-P there are thirty triggers^21^ divided into five domains; Treatment (12 triggers), Drugs (1 trigger), Coercive Treatment (4 triggers), Medications (7 triggers) and Continuity and transitions (6 triggers).

### Sample and data collection

We analyzed medical records from the inpatient psychiatric department at Nord-Trøndelag Hospital Trust.

All patients aged 65 or older admitted to psychiatric hospital care in the period from January 1^th^ 2022 to December 31^th^ 2023, who did not decline the review of their patient hospital records, were included in the study (Table 1).

**Table 1.** Characteristics of study participants; sex, age and treatment unit, data on education and civil status.

	Study cases
Patients in total	184 (100%)
Sex	
Men	72 (39%)
Women	112 (61%)
Age	
<= 64	0
65–69	51 (28%)
70–74	60 (33%)
75–79	35 (19%)
80–84	21 (11%)
85–89	12 (6.5%)
90–94	3 (1.5%)
>95	2 (1%)
Education	
Not finished compulsory school	n.a
High school	22 (12%)
Upper secondary school	31 (17%)
University colleges/University	35 (19%)
Missing	96 (52%)
Civil status	
Married/cohabiting	87 (47%)
Single	97 (53%)
Treatment unit	*n* = 245
Acute unit	58 (24%)
Short-term unit	93 (38%)
Long-term unit	94 (38%)

Treatment unit.

Short-term units = general psychiatric units, Substance use and addiction units.

Long-term units = units for psychosis, eating disorders, old age psychiatry, secure units.

The scope of psychiatric care includes the Interdisciplinary Specialized Treatment for Drug Abuse, thus the dataset pertains to both patients undergoing treatment for substance misuse and those receiving psychiatric care.

The patient hospital records were analyzed using the GTT-P by the investigation teams comprising a psychiatrist and three psychiatric nurses in the hospital trust. Each record was analyzed by the psychiatrist and two of the three nurses. The investigation team comprised experienced health professionals with prior expertise in psychiatric care for the elderly where two of the nurses participated in the 2017 GTT-P investigation team.^21^ Before commencing the analysis, all team members underwent training under the guidance of the project leader (A.O). The training consisted of introduction to the method and a calibration exercise where the team members reviewed the same five records. This was followed by a discussion and comparison of the results, which demonstrated consistency in how the definition of an AE and triggers were interpreted and practiced. Additionally, two further joint meetings with the team were held to ensure a shared understanding of the research procedures.

Two nurses independently reviewed each record in groups of twenty before comparing their results and validating their findings with the psychiatrist. The psychiatrist also specifically reviewed the medication administration in all patient hospital records. Through discussion, the team reached consensus. In cases of disagreement, the psychiatrist’s decision was final.

Given that some team members were initially “beginners” and reviewed records from a complex group, they initially exceeded the set time limit of 30 min per record. By the end of the project, they averaged 30 min per record. The identified AEs were classified by type and severity was rated. The team also had the opportunity to discuss with the project leader when necessary.

The region, from which the patients originate, maintains a relatively stable population of approximately 130,000 inhabitants. This predominantly rural area lacks large cities but remains fairly representative in terms of geography, economy, employment types, age distribution, morbidity, and mortality in Norway.^24^

Primary diagnosis for each patient was also identified in the review of the patient records (see Table 2).

**Table 2.** Main diagnoses of study participants (ICD-10 codes).

	Diagnoses	Study cases n, %
F00-09	Organic, including symptomatic, mental disorders	24 (13.0)
F10–19	Mental and behavioral disorders due to psychoactive substance use	23 (13.0)
F20–29	Schizophrenia, schizotypal, delusional disorders	16 (8.7)
F30–39	Mood [affective] disorders	68 (37.0)
F40–48	Neurotic, stress-related and somatoform disorders	22 (12.0)
F50–59	Behavioral syndromes associated with physiological disturbances and physical factors	1 (0.5)
F60–69	Disorders of adult personality and behavior	4 (2)
F90–98	Behavioral and emotional disorders with onset usually occurring in childhood and adolescence	1 (0.5)
F70–79, 99	Others	24 (13)
G00–99	Diseases of the nervous system	1 (0.5)
R00–99 others	Symptoms, signs and abnormal clinical and laboratory findings not elsewhere classified	
Sum		184 (100)

### Statistical analysis

Descriptive statistics are presented using frequencies and relative frequencies for categorical variables. Differences in AE occurrence (yes/no) between sexes were examined using a Chi-square test. Mann-Whitney U test was used to compare age between patients with one or more AE to those without AE, and number of triggers identified in patient records with and without AEs. Fisher’s exact test was used to compare the occurrence of specific triggers between patient records with and without AE. To determine the association between triggers categorized into domains and AEs we used multivariate mixed model logistic regression with AE (yes/no) as the dependent variable, and presence of specific trigger domains (yes/no) as independent variable, and patient-ID as random effect to account for repeated observations. *p*-values below 0.05 were considered as statistically significant. Data analysis was performed in Rstudio with R version 4.4.0 using package “ggplot2” for visualizations and function “glmer” from the package “lme4” for linear mixed models.

## Results

### Patient characteristics and adverse event distribution

In total, 245 patient admissions, encompassing 184 unique individuals were included in the study cohort (see Table 1). Most had a single hospital admission during the study period, while a few had multiple admissions, with one patient admitted seven times. AEs were identified in 20 patients (10.9%), with three individuals experiencing multiple events. Patients who experienced AEs were significantly older (median age 76) compared to those without any recorded event (median age 71, *p* = 0.005). No significant association was found between sex and AE occurrence (*p* > 0.99).

### Types and severity of adverse events

**Figure 1.**
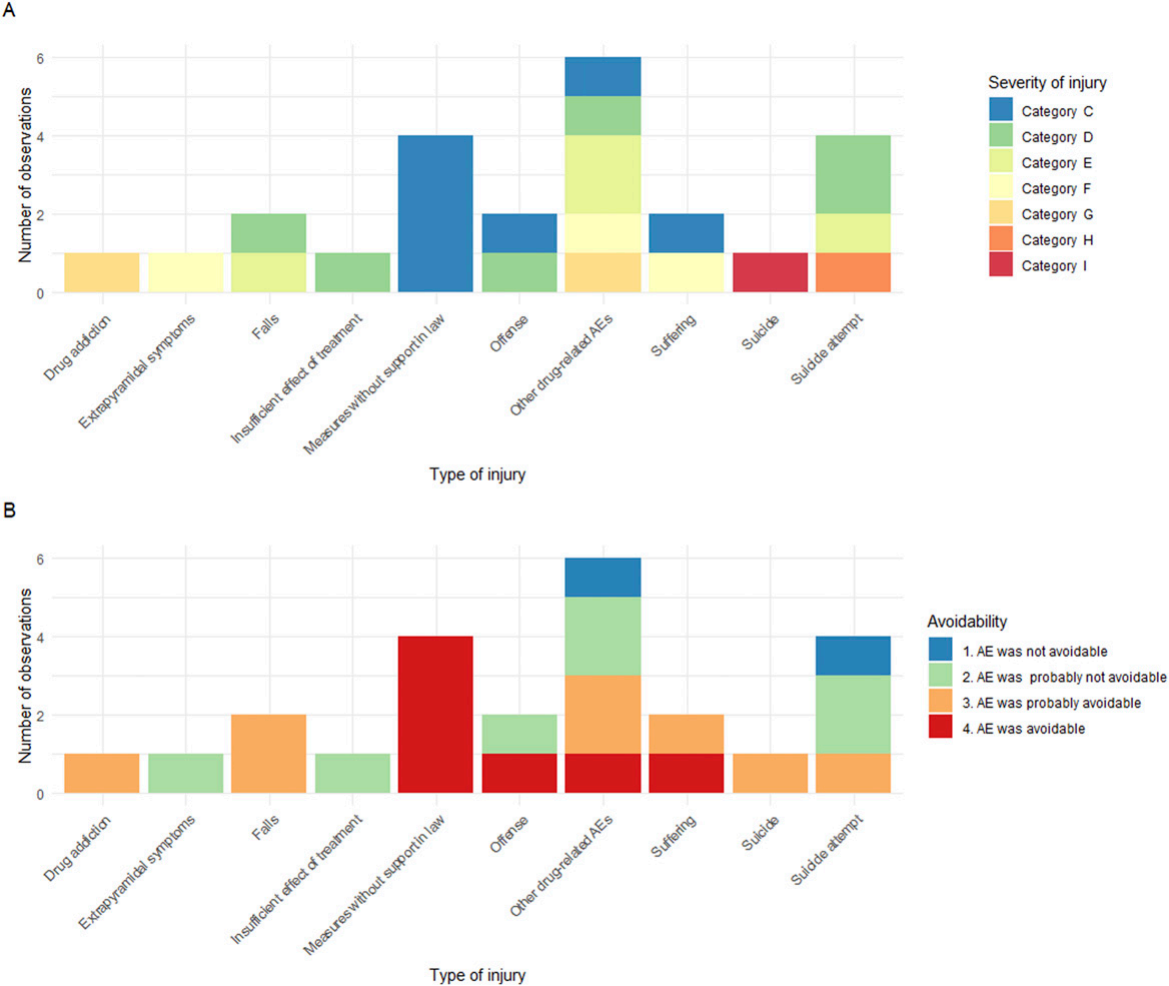
Overview of the adverse events observed in the study cohort. (a) Adverse events colored by severity of injury, where category C is the least severe and category I is the most severe. (b) Adverse events colored by degree of avoidability.

Among the 24 AEs recorded, the most frequent were drug-related complications (*n* = 6), followed by measures without support in law (*n* = 4), suicide attempts (*n* = 4), and psychological distress or suffering (*n* = 2). One case involved completed suicide, classified as a catastrophic event. AE severity was assessed using standardized harm categories: 13 events were non-harmful (C/D), 7 were moderate (E/F), 3 were severe (G/H), and 1 was fatal (I). A majority (*n* = 15) were deemed potentially avoidable (Figure 1).

### Trigger occurrence and association with AE

The review process identified 901 triggers across the patient records, with triggers present in 96% of cases. Patient records with AEs had a significantly higher number of triggers (median: 7) compared to those without (median: 3), (*p* < 0.001). No AE was documented in the absence of an associated trigger. The most prevalent triggers were lack of designated care coordination (52.7%), change of treatment unit (46.9%), and Consultation with a physician on call/physician from another specialty (46.1%). The patient-responsible physician is designated as the principal medical coordinator, ensuring continuity of care, facilitating interdisciplinary collaboration, and serving as the consistent point of contact for the patient throughout their engagement with specialist health services.^25^ Table 3 shows the distribution of the triggers in the sample.

**Table 3.** Triggers identified in examined records with and without adverse events (AE) total of examined records: *N* = 245. Triggers as defined in the Norwegian handbook (Okkenhaug et al., 2019).

Triggers		Patient records with no AE (*n* (%))	Patient records with AE (*n* (%))	*p-*value
Treatment				
B1	Absence of treatment plan	13 (5.9%)	0	0.623
B2	Absence of individual plan	1 (0.5%)	0	1
B3	Lack of review of suicide risk	3 (1.4%)	0	1
B4	Consultation with a physician on call/physician from another specialty	98 (44.3%)	15 (62.5%)	0.130
B5	Change of diagnosis	13 (5.9%)	3 (12.5%)	0.197
B6	Self-harm	3 (1.4%)	5 (20.8%)	**<0,001** ^**^
B7	Undesired effect of treatment	5 (2.3%)	4 (16.7%)	**0,006** ^*^
B8	Threats, violence and inappropriate behavior	26 (11.8%)	6 (25.0%)	0,102
B9	Increased surveillance	17 (7.7%)	5 (20.8%)	0.049*
B10	Lack of somatic status	29 (13.1%)	0	0.088
B11	Absence of contact with relatives	26 (11.8%)	1 (4-2%)	0.489
B12	Others	88 (39.8%)	11 (45.8%)	0.663
Drugs				
R1	Lack of examination of substance abuse	28 (12.7%)	5 (20.8%)	0.339
Coercive treatment				
T1	Coercion	47 (21.3%)	11 (45.8%)	0.011*
T2	Coercion treatment – administrative failure	4 (1.8%)	5 (20.8%)	**0.001** ^**^
T3	Conversion from voluntary treatment to coercion (emergency law)	2 (0.9%)	3 (12.5%)	**0.007** ^*^
T4	Police assistance	8 (3.6%)	0	1
Medicine				
L1	Use of three or more different antipsychotic drugs	0	0	n.a
L2	Treatment with anticholinergics	4 (1.8%)	3 (12.5%)	0.022*
L3	More than four different psychofarmaca	7 (3.2%)	1 (4-2%)	0.567
L4	Two or more benzodiazepine or treatment more than 3 months	32 (14.5%)	6 (25.0%)	0.229
L5	Metabolic risk factors	0	0	n.a
L6	Guidelines not followed when medicine require regular tests	13 (5.9%)	5 (20.8%)	0.021*
L7	Medication, others	7 (3.2%)	9 (37.5%)	**<0,001** ^**^
Continuity and transition				
K1	Unplanned inpatient treatment or contact with psychiatric acute unit	49 (22.2%)	7 (29.2%)	0.447
K2	Reinstatement within 30 days	16 (7.2%)	6 (25.0%)	0.012*
K3	Change of treatment unit	98 (44.3%)	17 (70.8%)	0.017*
K4	Unplanned discharge or ending outpatient treatment	7 (3.2%)	0	1
K5	Lack of doctor`s round during the last 12 months	0	0	n.a
K6	Absence of patient-responsible physician or coordinator	117 (52.9%)	12 (50.0%)	0.832

Statistically significant p-values (<0.05) are shown in bold. *p-value <0.05, **p-value <0.001.

### Clinical diagnoses and AE distribution

Mood disorders were the most common diagnosis (37%) and were disproportionately represented among patients with AEs (60%) (see Table 2). Organic mental disorders comprised 25% of AE cases, with a subset of these patients experiencing multiple events. Anxiety and substance use disorders were less frequently linked to AE occurrence. The distribution underscores potential diagnostic-specific vulnerability.

### Trigger categories and risk association

Triggers related to compulsory care measures (OR 3.30; 95% CI: 1.01–10.85; *p* = .049) and medication processes (OR 8.11; 95% CI: 1.59–41.39; *p* = .012) were significantly associated with elevated AE risk (Table 4).

**Table 4.** Triggers associated with adverse events.

Trigger domain	OR (95% CI)	*p-value*
Treatment	2.41 (0.34–16.87)	0.370
Coercive treatment	3.30 (1.01–10.85)	0.049*
Continuity and transition	6.36 (0.72–56.13)	0.096
Drugs	2.57 (0.41–16.23)	0.320
Medicine	8.11 (1.59–41.39)	0.012*

Statistically significant p-values (<0.05) are shown in bold. *p-value <0.05.

## Discussion

### Statement of the principal findings

In this study AEs occurred at least once in 20 of the older inpatients and this equates to approximately 10.9%. Of 24 identified AEs, 15 could have been prevented if appropriate measures had been implemented when the patient interacted with the healthcare system. The most common AE was “other drug-related AE” followed by “measures without support in law” and “suicide attempt.”

### Strengths and weaknesses of the study

The Department of Psychiatry at Nord-Trøndelag Hospital Trust was certified according to ISO standards from 2005 to 2018. This certification involved systematic oversight of records by unit leaders and audit teams.^26^ Such practices are not common in Norwegian psychiatric clinics, which may render the department not representative for other psychiatric departments in terms of quality systems. Additionally, there is a recognized limitation in retrospective chart reviews regarding the potential to overlook AEs that are not documented in the records.

The clinical sample in this study was relatively small, which increases the risk of reduced external validity and generalizability. This single-center design in this article presents the results from a dataset obtained from a single hospital trust with a limited number of AEs, which limit generalizability.

A relevant limitation of the study is the inherent subjectivity involved, particularly in assessing the “avoidability” of AEs, despite the use of a team-based consensus approach. While “severity” is more clearly defined according to the NCC MERP classification, the determination of “avoidability” allows for greater interpretative discretion.

Utilizing 30 distinct triggers, we employed data reduction techniques to develop a model that could be estimated and replicated more broadly within Norway. Ideally, cluster analysis techniques should be applied when analyzing these data. The rationale is that the grouping we have applied may not optimally predict AEs. It is possible that specific combinations of different triggers could provide a more accurate prediction. Our initial intention was to use cluster analysis or similar data reduction techniques to identify such groups. However, the number of observations and AEs in this dataset is insufficient relative to the number of triggers, precluding such an analysis. Scaling up the project would be necessary to achieve this.

### Discussion in relation to other research

Eggenschwiler et al.,^16^ found in a systematic review, that older patients, in an general patient cohort, have a higher incidence of AEs compared to younger patients. In our study we found an AE in 10.9% of older inpatients, compared to 7.9% of adult inpatients of all ages in a previous study.^21^ However, the level of AEs in our study differs compared other similar studies from the psychiatric specialist health care which reported that approximately 17-28% patients experienced a patient safety event.^18,19^ Comparative data from the somatic healthcare sector for the adult population in Norway demonstrate that, in this and in our earlier study, the measured outcomes appear to be lower than those observed in the general somatic population.^21^ In 2023, AEs were reported in 12.0% of somatic hospitalizations.^27^ One possible contributing factor to the higher incidence of harm observed among older adults in mental health care may relate to the presence of more somatic comorbidities in this population, potentially aligning their risk profile more closely with that observed in somatic healthcare settings.

Our findings indicate that 15 out of 24 identified AEs could have been prevented if appropriate measures had been implemented when the patient interacted with the healthcare system. Examples of measures to prevent drug-related AEs and/or fall injuries may include reducing high doses of benzodiazepines and increasing vigilance regarding the anticholinergic effects of prescribed medications. This is consistent with previous research.^4,28,29^

Compared to Okkenhaug et al.,^21^ which included a random sample of adults across all age categories and also included outpatient patients, there is a shift from “psychological AEs” and “treatment-related AEs” to “drug-induced AEs.” Drug-related AEs (extrapyramidal side effects, drug addiction and others) represent a serious and frequently occurring risk area for elderly patients in psychiatry.^4,30^

Due to a higher rate of frailty among older patients in psychiatric care, these patients may also be more prone to longstanding effects of AEs.^31^ Lack of knowledge among healthcare professionals, lack of review of drug charts, burden of multimorbidity, enhanced pain, polypharmacy and drug-dependence has been described as risks in earlier research.^3,29,32,33^ Even without the elderly as the primary case group, several studies in psychiatry describe drug-related AEs as the most frequently observed form of harm.^19,34,35^ In Marcus`s survey,^15^ the most frequent events were medication errors (delayed and missed doses, 17.2%), followed by adverse drug events (4.1%), falls (2.8%), and assaults (1.0%). Most reported patient safety events (94.9%) resulted in little or no harm although more than half of the events (56.6%) were deemed preventable.^15^

Our study identified multiple instances of suicidal behavior with varying degrees of severity, which is consistent with findings reported in previous research. Suicide and self-harm among older adults is an escalating concern, driven by a growing elderly population and multiple age-related risk factors.^7,36^ Risk factors includes physical illness, familial issues, financial issues, hopelessness and living in rural areas where more likely to experience social isolation among others.^8^ Research indicates that, compared to younger adults, suicidal older adults are significantly more likely to be discharged from emergency departments without receiving a mental health evaluation.^37^

The most commonly identified issues documented in “other treatment-triggers” included: incomplete or absence of discharge medical report and other relevant medical records; lack of routine monitoring of body weight and laboratory testing; prolonged hospitalization due to underlying somatic conditions; and insufficient implementation of fall prevention strategies. The findings of most frequent triggers are not consistent with Nilsson et al.^18^ and Okkenhaug et al.,^21^ where the absence of a treatment plan was the most common trigger.

Compared with result from Okkenhaug et al.^20^ and Nilsson et al.^18^ the older adults experienced more often change of treatment unit. This is consistent with Conlon et al.^38^ findings, which indicate that the geriatric cohort is more likely to experience care transition between medical and psychiatric hospital wards. Care transitions increase the risk of service duplication, conflicting recommendations, and medication errors.^38^

Our study demonstrates a correlation between the number of identified triggers and AEs. Records with a detected AE contained an average of 7 triggers, compared to an average of 3 triggers in records without an identified AE. The greater the number of triggers found in a patient record, the higher the risk of the patient experiencing an AE. This finding aligns with previous data from the same department^20,21^ and other research in somatic care.^39,40^ To our knowledge, aside from Okkenhaug et al.^21^ this correlation has not been previously discussed in the context of psychiatric care.

### Implications for clinicians and policymakers

When adapting GTT for psychiatric care, it is reasonable to consider potential synergies in care delivery approaches. Triggers such as “lack of a treatment plan” or “undesired effects of treatment,” which we identified as significant in psychiatric care, may also be pertinent to the somatic GTT. If an examination of a patient record reveals that a patient was restrained without formal authorization, prescribed high doses of Z-drugs, and exhibiting shuffling gait, constipation, and catheterization needs, may suggest two plausible AEs; “Measures without support in law” and “other drug-related AE.”

While applying the GTT in psychiatric inpatient care can help identify areas for safety improvement, integrating the GTT-P through stakeholder engagement promotes a stronger safety culture and enhances care quality.^41^

## Conclusions and need of further research

This study identifies a strong link between specific clinical triggers—particularly pharmacological factors, care transitions, and discontinuity in responsibility—and AEs in older psychiatric inpatients. Frailty and multimorbidity heighten vulnerability in this group, emphasizing the need for proactive risk identification and structured care models to reduce preventable harm. Improved AE detection may support safer clinical pathways and better outcomes Supplemental Material 1.

To our knowledge, this is the first application of the GTT in geriatric psychiatry. While prior studies have focused on general adult populations, this work addresses a critical gap.^34,35,42^ Despite limitations in sample size and setting, the findings warrant broader validation and refinement of the GTT-P. Emerging methods like automated trigger detection offer promising avenues for scaling and enhancing patient safety in mental health care.^43^

## Supplemental Material


Supplemental material - Mitigating risk in psychiatric hospital care for older adults by identifying adverse events with Global Trigger Tool for psychiatric patients
Supplemental material for Mitigating risk in psychiatric hospital care for older adults by identifying adverse events with Global Trigger Tool for psychiatric patients by Arne Okkenhaug, Eivind Aakhus, Guro F. Giskeødegård, Bodil J. Landstad and Ellen T. Deilkås in International Journal of Risk & Safety in Medicine
