# Supplementary material for: Mitigating risk in psychiatric hospital care for older adults by identifying adverse events with Global Trigger Tool for psychiatric patients
Source: Int J Risk Saf Med. 2026 Jan 11;37(3):328–37. doi: 10.1177/09246479251409028 (PMC13428814; doi:10.1177/09246479251409028)
Supplement: Supplemental material - Mitigating risk in psychiatric hospital care for older adults by identifying adverse events with Global Trigger Tool for psychiatric patients [file sj-pdf-1-jrk-10.1177_09246479251409028.pdf]

Manuscript ID JRK-25-0003. R1

Dear reviewers,

Thank you for recommended publication and giving us the opportunity to re-submit a revised draft of our manuscript for publication in JRK. We really appreciate the time and effort that you dedicated to providing feedback on our manuscript, and we are grateful for the insightful comments on and valuable improvements to our paper. We have carefully considered the comments and tried our best to address every one of them. Please see below, a table with responses to the reviewers' comments and concerns. The revisions are highlighted in red.

Many thanks,

The authors

No	Comments	Comments from authors	Heading	Page (manuscript with changes in red)
	**Reviewer 1**			
1.	Avoidability Criteria:<br>The criteria for judging an AE as "avoidable" are central to the conclusions but are not explicitly defined in the methods. Please provide a brief description of the operational criteria used by the review team (e.g., based on deviation from guidelines, clear error in judgment, or absence of a standard procedure).	Based on a validated handbook for GTT-P, we have added a sentence to clarify the criteria for determining AEs as avoidable.	Methods, starting point for the study	Page 4
2.	Sample Characterization:<br>While the setting is described as "fairly representative," a brief comment on the socio-demographic or clinical characteristics of the population compared to national averages would strengthen the argument for generalizability.	We agree with the reviewer's conclusion; however, it would be a substantial undertaking for us to obtain valid data comparing our population with national data on psychiatric inpatients over the age of 65. Nevertheless, this is a valuable suggestion that we will		

Åpen

		consider for future research.		
3.	Timeline Clarification:<br>The abstract states "between 2022–2023," while the methods specify "January 1st 2022 to December 31st 2023." Please ensure consistency throughout the manuscript.	To avoid exceeding the journal's word limit for the abstract, we chose to use 'between 2022–2023' in that section, while providing a more precise specification of the time period in the Methods chapter.	Methods, Sample and Data Collection	Page 5
4.	Limitations:<br>The limitations section could be expanded. Key points to address more explicitly include:<br>The single-center design and relatively small sample size, which limit generalizability.<br>The potential for retrospective chart reviews to miss AEs that are not documented.<br>The inherent subjectivity in determining AE "avoidability," despite the team-based consensus approach.	We have re-written the limitation section according to the comments.	Discussion,<br>Strengths and weaknesses of the study	Page 10-11
5.	Language:<br>Ensure consistent use of "GTT-P" after its first definition. A few sentences, particularly in the abstract and introduction, are long and complex. Breaking them down could improve readability.	We have gone through the paper to ensure consistent use of GTT-P and changed long sentence. We have also ensured consistent use of AE.		
	**Reviewer 2**			
1.	Abstract:<br>In the background you state that it is important to validate an earlier version of the GTT-P to include old psychiatric patients. This is confusing and not further addressed. What do you mean by "earlier version"? Concerning "old" patients – were they not included in the GTT-P? Also, this is not fully coherent with the concise main objective.	We have change from "earlier" to "previous version" and re-written the introduction to substantiate the paragraph in the background	Abstract and the Introduction	Page 2-4
2.	Introduction, page 3, lines 43-50:<br>As the specific AE definition for psychiatric patients is given elsewhere, I	We have deleted the sentence	Introduction	

Åpen

	find these lines unnecessary.			
3.	Methods, page 5, line 22:<br>You refer to Table 3 as the first reference to a table. I leave to the journal to judge if this is correct. Alternatively, you could leave out the table reference.	We are agreed, we leave to the journal to judge.	Methods	
4.	Sample and data collection, page 5, lines 28-31 until individuals, and Table 1: This is a result!	We have re-written the sentence and removed a part to the first sentence in result.		Page 5 and 8
5.	Sample and data collection, page 5 line55 to page 6 line 8:<br>The writing is confusing to me. How many team members were involved totally?	We have re-written the chapter and hope it becomes clearer		Page 6
6.	Statistical analysis, page 7, line 16. I guess a "period" is missing between "variables" and "differences".	Yes, thanks a lot.		
7.	Page 9, Trigger Categories and Risk Association, lines 41-45: These non-significant results need not be mentioned here.	We have removed the sentence.		
8.	The discussion part should benefit from being shortened and more focused.			
9.	Discussion, page 10, first paragraph:<br>Start the discussion with your principal findings and delete this paragraph or incorporate small essential parts of it in the discussion.	We have deleted the paragraph and started with the principal findings.		
10	Discussion, page 10, statement of the principal findings:<br>You need not repeat the exact numbers now given in brackets.	We have deleted		
11	Discussion, page 11, line 45:<br>Please specify in the text regarding ref #16 and #23 if they refer to a psychiatric or general patient cohort. This would make the reading easier.	We have re-writted the sentence.		Page 11
12	Discussion, page 11, line 57:<br>The expression "our studies" is not fully clear. Please rewrite and give a reference to #23.	We have changed the sentence and referred to #23		Page 11
13	Discussion, page 12, lines 18-23:<br>You need not repeat the most common AEs including exact numbers.	We have deleted the sentence.		
14	Discussion, page 12, lines 32-46:<br>I suggest that the full paragraph is deleted	We have removed the paragraph.		

Åpen

15	Discussion, page 12, lines 50-56:<br>rewrite, not necessary to repeat exact finding from the results section.	We have deleted the sentence.		
16	Discussion, page 14, first paragraph:<br>I suggest substantial shortening and not repeating findings from the results section.	We have shortened and re-written the paragraph.		Page 13
17	Discussion, page 15, Implications for clinicians and policymakers:<br>Suggest that the last paragraph “Physicians and nurses…..” is deleted.	We have deleted the paragraph.		
18	Conclusions and need of further research:<br>Should be substantially shortened to include 1-2 paragraphs	We have re-written the chapter.		Page 14-15
19	Table 2:<br>Suggest writing “Main diagnoses of…” in the heading	We have changed the heading		Table 2
20	Table 3:<br>I guess that p-values should be < 0.001, not >0.001	Yes, thanks a lot.		

Åpen
